# Novel Semi-Parametric Algorithm for Interference-Immune Tunable Absorption Spectroscopy Gas Sensing

**DOI:** 10.3390/s17102281

**Published:** 2017-10-07

**Authors:** Umberto Michelucci, Francesca Venturini

**Affiliations:** 1 Udata.Science, Dübendorf 8600, Switzerland; um@udata.science; 2 Institute of Applied Mathematics and Physics, Zurich University of Applied Sciences, Winterthur 8401, Switzerland

**Keywords:** interference, interference cancellation, noise reduction, digital filtering, spectroscopy, sensors

## Abstract

One of the most common limits to gas sensor performance is the presence of unwanted interference fringes arising, for example, from multiple reflections between surfaces in the optical path. Additionally, since the amplitude and the frequency of these interferences depend on the distance and alignment of the optical elements, they are affected by temperature changes and mechanical disturbances, giving rise to a drift of the signal. In this work, we present a novel semi-parametric algorithm that allows the extraction of a signal, like the spectroscopic absorption line of a gas molecule, from a background containing arbitrary disturbances, without having to make any assumption on the functional form of these disturbances. The algorithm is applied first to simulated data and then to oxygen absorption measurements in the presence of strong fringes.To the best of the authors’ knowledge, the algorithm enables an unprecedented accuracy particularly if the fringes have a free spectral range and amplitude comparable to those of the signal to be detected. The described method presents the advantage of being based purely on post processing, and to be of extremely straightforward implementation if the functional form of the Fourier transform of the signal is known. Therefore, it has the potential to enable interference-immune absorption spectroscopy. Finally, its relevance goes beyond absorption spectroscopy for gas sensing, since it can be applied to any kind of spectroscopic data.

## 1. Introduction

Due to the enormous progress in availability and performance of laser light sources and electro-optical components, tunable diode laser absorption spectroscopy (TDLAS) has entered various disciplines both in research and industrial applications. Being a highly-sensitive, selective, fast, non-destructive and in situ method, TDLAS is currently more and more used for quantitative assessment of gas concentration in several fields. These include, to mention only a few, atmospheric environmental monitoring [1,2,3,4,5,6], medical diagnostics [7,8,9], chemical analysis [10], and industrial process control [11,12,13]. The increasing number of applications has pushed the requirements for this method both in terms of sensitivity and in terms of stability. On the other hand, for practical and commercial applications, there is a growing interest in compact, simple in design and cost-effective sensitive sensors that do not require special optical components but guarantee the sensitivity achievable with complex laboratory equipment.

One of the most common limits to the sensor performance is the presence of unwanted interference fringes due to etalons [14]. These interferences may arise due to multiple reflections from reflecting or scattering surfaces in the system, like mirrors, lenses, optical fiber end faces, laser-head windows or dust particles in the gas [14]. Even surfaces that are diffusive, for example due to dust deposited on sensor windows, can give rise to fringes over time [15]. In particular, fringes that have a free spectral range (FSR) on the order of the width of the absorption lines contribute to significant errors in the determination of the line features. Special strategies have been proposed to deal with these types of interferences, as, for example, in [16], where for small fringe amplitudes and a measurement window that is a multiple of the fringe FSR, the fringe was removed by an analysis in the Fourier domain. Furthermore, since the amplitude and the frequency of these interferences depend on the distance and alignment of the optical elements, they are affected by temperature changes and by mechanical disturbances, giving rise, for example, to a drift of the output signal, thus worsening the long-term performance of the system [17].

The simplest strategy to reduce the effects of interference fringes consists of using anti-reflection coating and wedging or angling of the optical surfaces. Other approaches include, to mention only few, dithering one of the surfaces creating the interference and integrating the signal so as to average out its influence [18,19], selecting a particular modulation frequency [20] or modulation scheme [21,22], specifically choosing the distance between the interfering surfaces [23], and post-processing filtering [24]. A comprehensive review on signal enhancement and noise reduction techniques can be found in [25]. Although all of these approaches have been successfully implemented in past years, they nonetheless have limitations in the practical implementation depending on the application-specific conditions. For example, it may not always be possible to dither one surface, and the application of a specific detection scheme may limit the flexibility of the measurement.

In this work, a new and widely usable approach is presented that relies only on post processing of the data. Therefore, it requires no modification to the apparatus setup or hardware, and can be easily adapted to different experimental configurations. The new presented algorithm allows the extraction of a signal, as the absorption lines of a gas molecule, from a background containing arbitrary disturbances without having to make any assumption on the characteristics of these disturbances in terms of functional form. Therefore, it has the potential to improve the sensitivity and the stability of TDLAS. Furthermore, this algorithm, which is particularly easy to implement if the Fourier transform of the signal can be written in closed form, is very general and can be applied to any kind of spectroscopic data. The paper is organized as follows: Section 2 describes the fundamentals of the method and of the algorithm; Section 3 demonstrates its application to simulated signals; and Section 4 shows the results for a case of direct absorption spectroscopy for oxygen gas sensing.

## 2. Description of the Algorithm

The algorithm described in this work has the ability to identify a spectroscopic feature from an arbitrary background that does not need to be modeled. The total signal detected in an experiment, here referred to as “total signal” $I_{tot}\left(x\right)$, is modeled as a sum of two contributions: one spectroscopic feature, like an absorption line, referred to here as “signal” I(x) and a background referred to here as “background” B(x):

(1) $$I_{tot}\left(x\right)=I\left(x\right)+B\left(x\right).$$

In the case of direct absorption spectroscopy, I(x) is the absorbance and $I_{tot}\left(x\right)$ is the distorted absorbance due to B(x). As mentioned above, the method shines if the background B(x) cannot be modeled by a known analytical expression. In fact, if B(x) is not known, it is not possible to perform a nonlinear fit of $I_{tot}\left(x\right)$ and extract the signal I(x) without making assumptions on the functional form of B(x). On the other hand, if the functional form is known but very complex, the algorithm may be advantageous because the inclusion of the background in the nonlinear fit may not be possible. Another significant advantage of the proposed algorithm is that the extraction works equally well independently of the amplitude of the interferences, as it will be shown in Section 3 and Section 4.

### 2.1. Steps of the Algorithm: General Description

Before describing the steps of the algorithm, the nomenclature and hypothesis for its applicability are introduced. The algorithm is based on the main hypothesis that the Fourier transform of the background B(x) is significantly different than zero only for values of *k* smaller than a certain cut-off $k_{0}$

(2) $$F\left(I_{tot}\right)\left(k\right)=\left\{\begin{array}{c} F\left(I\right)\left(k\right)+F\left(B\right)\left(k\right)\mathrm{for}|k|<k_{0},\\ F\left(I\right)\left(k\right)+\epsilon \mathrm{for}|k|>k_{0}, \end{array}\right.$$

where F(·) denotes the continuous Fourier transform (CFT), $k_{0}$ a cut-off frequency and ϵ contains the contribution of F(B)(k) for $|k|>k_{0}$, which is assumed to be negligible. One central aspect is the determination of a reasonable estimate for this cut-off frequency, as described in the section “Determine the cut-off frequency”.

Note that this formulation applies to continuous functions. Since in practice there will always be only a discrete set of points, it is necessary to approximate the CFT in Equation (2) by a modified discrete Fourier transform (DFT)

(3) $$D_{i}\left(I_{tot}\right)=\left\{\begin{array}{c} D_{i}\left(I\right)+D_{i}\left(B\right)\mathrm{for}\left|i\right|<i_{0},\\ D_{i}\left(I\right)+\epsilon \mathrm{for}\left|i\right|>i_{0}, \end{array}\right.$$

where $D_{i}\left(\cdot \right)$ denotes the modified DFT defined in Appendix A Equation (A6), $i_{0}$ the cut-off point corresponding to the cut-off frequency $k_{0}$, and ϵ contains the contribution of $D_{i}\left(B\right)$ for $|i|>i_{0}$, which is assumed to be negligible.

**Figure 1:**
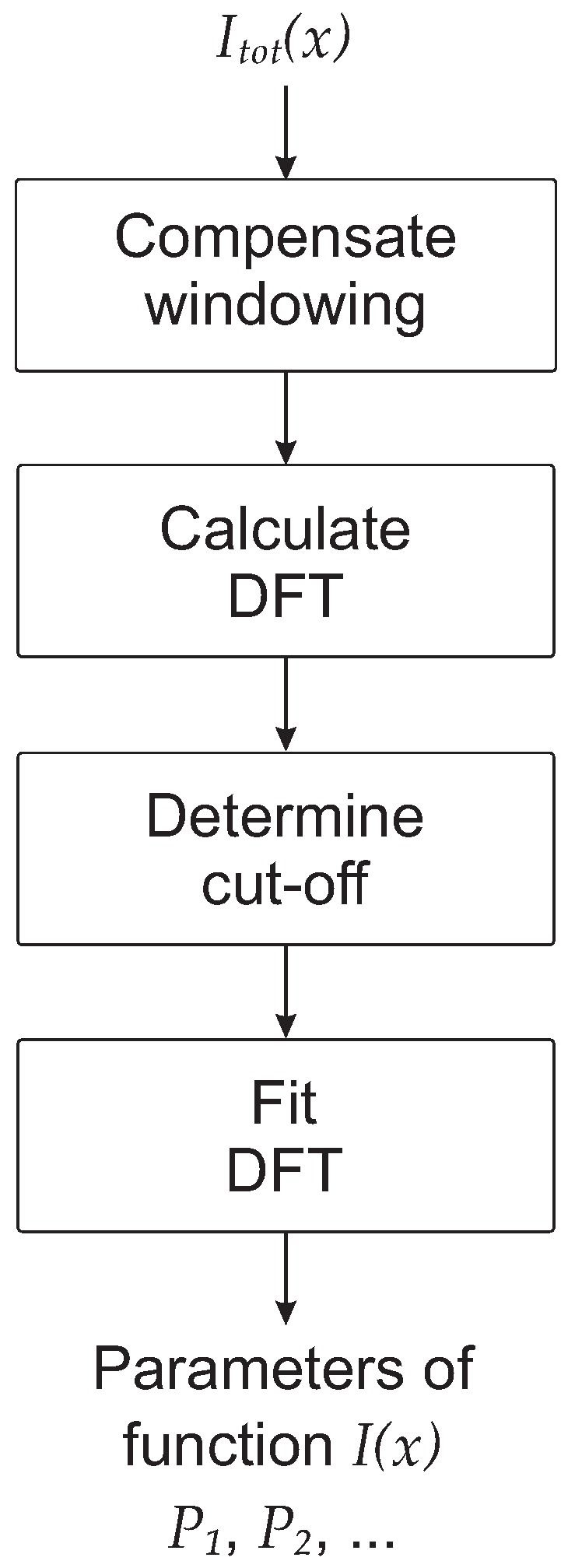
Schematic flow diagram of the steps of the algorithm to extract a signal I(x) from a total signal $I_{tot}\left(x\right)$.

The schematic flow diagram of the algorithm is shown in Figure 1 to give the reader a high-level understanding of the idea behind it. The single steps are described in detail below.

### 2.2. Compensate Windowing

In all experiments, the data always cover a limited range in the *x*-direction. For example, the data shown in this paper are measured for a finite laser wavelength range. Mathematically, this is equivalent to applying a rectangular window (RW) before calculating the Fourier transform. This is a problem that, if not addressed, will limit the precision that can be achieved with the described algorithm. In fact, the RW results in making ϵ in Equation (2) not negligible anymore, and thus leads to an error in the fitting procedure of |F(I(x))(k)|. This is due to the fact that the DFT of a RW has an amplitude envelope that is proportional to 1/k, and so it does not go to zero fast enough [26].

To reduce the effect of windowing considerably, the proposed algorithm applies a more intelligent window. The not so often used Tukey window [27,28] has remarkable properties that help tremendously in reducing ϵ dramatically. The Tukey window, indicated with T(x), is a perfectly flat (constant) symmetric function in the middle that then decreases rapidly to zero on the sides.

**Figure 2:**
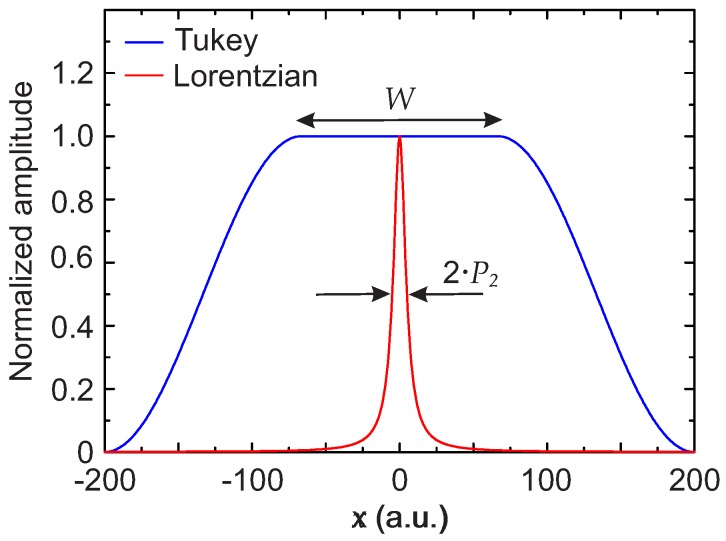
Schematic representation on how to choose the width of the Tukey window *W* compared to the Lorentzian HWHM $P_{2}$. Here, it is $W=20\;P_{2}$.

The width of the constant part of the Tukey function has to be chosen intelligently. Figure 2 shows a Lorentzian function with a half width at half maximum (HWHM) indicated as $P_{2}$ and Tukey function with a width indicated as *W*, with both functions normalized to 1 for clarity. In this work, a.u. indicates arbitrary units. As it is easy to understand from Figure 2, if *W* is significantly bigger than $P_{2}$, then T(x)I(x) can be approximated with I(x). Therefore, defining ${\tilde{I}}_{tot}\left(x\right)=T\left(x\right)I_{tot}\left(x\right)$, it follows that

(4) $${\tilde{I}}_{tot}\left(x\right)=T\left(x\right)I\left(x\right)+T\left(x\right)B\left(x\right).$$

This is the function of which the DFT has to be calculated, instead of simply using $I_{tot}\left(x\right)$. Equation (4) can then be approximated under the assumption of *W* being significantly bigger than $P_{2}$ as

(5) $${\tilde{I}}_{tot}\left(x\right)=I\left(x\right)+T\left(x\right)B\left(x\right).$$

${\tilde{I}}_{tot}\left(x\right)$ is thus the sum of the signal of I(x) and a background that is the product of the original background B(x) and T(x). The modified background T(x)B(x) has a Fourier transform that goes to zero much more rapidly ([27,28]). This means that ϵ is much smaller if one considers ${\tilde{I}}_{tot}\left(x\right)$ instead of $I_{tot}\left(x\right)$. In other words, while using ${\tilde{I}}_{tot}\left(x\right)$, ϵ will contain the contribution of $D_{i}\left(T\cdot B\right)$ that is considerably smaller than $D_{i}\left(B\right)$ multiplied by an RW.

Analyzing the deviation between the output of the algorithm and the input signal I(x) with simulated data, it was established that the algorithm works well if the width of the Tukey window is $W≳20\;P_{2}$. In this work, $W=20\;P_{2}$ was used for both the simulated and for the experimental data. The case with $W=20\;P_{2}$ is shown schematically in Figure 2.

### 2.3. Calculate the DFT

The step after compensating for the windowing is the calculation of the DFT. To be able to extract the parameters of the function I(x) directly from the DFT, it is essential to approximate the CFT by a modified DFT as described in Appendix A. For all the data shown in this paper, the DFT was calculated using the formula (A6).

### 2.4. Determine Cut-Off

As shown in Figure 1, the next step is to determine the optimal cut-off point $i_{0}$ that plays an important role and needs to be chosen carefully. The approach proposed in this work is to choose $i_{0}$ so as to maximize the coefficient of determination $R^{2}$ obtained by fitting the DFT for $i>i_{0}$ to the functional form of the Fourier transform of the line shape. In our example for a Lorenztian function, the DFT was fitted by Equation (7), as is explained in more detail later. An example of the implementation is described in the following algorithm written in pseudo code, where N indicates the number of points of the dataset (i.e., the finite number of experimental points), D_i the ith point of the DFT of the signal ${\tilde{I}}_{tot}\left(x\right)$, Rsquared the coefficient of determination $R^{2}$, FFFT the functional form of the Fourier transform of I(x), and Rlimit the value which should be reached for $R^{2}$. This limiting value can be helpful since, beyond a certain value, even if $R^{2}$ continues to increase when increasing $i_{0}$, the quality of the fit will not improve significantly and it is not necessary to exclude more points from the DFT for the fit:

pointtoremove = 0
Rsqmin = 0
for i = 0 to N/2
 remove the first i points starting from D_0 to D_{i-1} from the DFT
 fit the remaining points D_i to D_{N/2} to the FFFT and save
  the fit parameter Rsquared
 if (i = 0) then pointtoremove = 0
 else if (Rsquared > Rsqmin) then pointtoremove = i and Rsqmin = Rsquared
 if (Rsqmin > Rlimit) then break loop
end of for loop.

**Figure 3:**
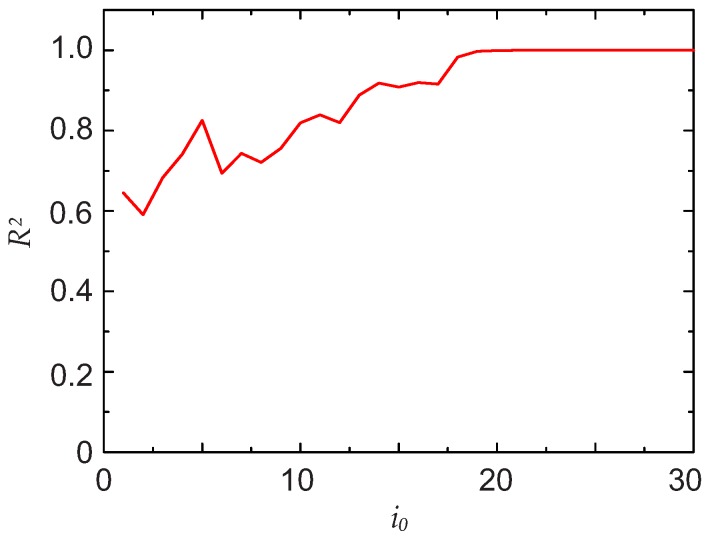
Evolution of $R^{2}$ with increasing value of the cut-off $i_{0}$. The data correspond to the third scenario described in Section 3.

At the end of this loop in the abovementioned pseudo-code $i_{0}$ is determined and saved in the variable pointtoremove. Rsquared can be chosen depending on the application. For the curves shown in this paper, the loop was stopped for Rlimit =0.99999. An example of the evolution of $R^{2}$ with increasing $i_{0}$ is shown in Figure 3. The data refer to the third scenario described later in Section 3. Above $i_{0}=30$, $R^{2}$ still continues to slightly increase, but the statistical goodness of the fit does not improve further.

### 2.5. Fit of DFT

The final step of the algorithm is to perform a nonlinear fit of the DFT for $i>i_{0}$. Since the functional form of the DFT is known, this is a standard procedure that can be performed, for example, with least-square-fit routines and will not be discussed here. After the fit I(x) is determined without the need of doing an inverse Fourier transform since the functional form of |F(I(x))(k)| is known.

### 2.6. Algorithm Applied to a Lorentzian Line Shape

In this section, the implementation of the algorithm for a Lorentzian signal I(x) is described as an example. This functional form was chosen because it describes the absorption lines of many gas molecules, as, for example, oxygen under atmospheric conditions. In other conditions, like at higher temperatures or lower pressures, the Gaussian contribution due to Doppler broadening cannot be neglected and a Voigt profile is a better description.

The Lorentzian function can be written as

(6) $$I\left(x\right)=\frac{P_{1}P_{2}}{\pi (x^{2}+P_{2}^{2})},$$

with $P_{1},P_{2}>0$. In this form, $P_{1}$ and $P_{2}$ represent the area and the HWHM of the line. Writing I(x) in this form is particularly advantageous since in direct absorption spectroscopy the gas concentration can be determined directly from the area under the line, and is thus directly proportional to $P_{1}$. In this formulation, |F(I(x))(k)| is then a simple exponential

(7) $$F\left(I\left(x\right)\right)\left(k\right)=P_{1}e^{-P_{2}\;\left|k\right|}.$$

Thus, once the parameters $P_{1}$ and $P_{2}$ are determined from the fit of the DFT, I(x) is known.

## 3. Application to Simulated Data

The novel algorithm was first applied to artificially simulated data to demonstrate its functioning and its performance. Since the signal to be extracted I(x) is known, it is possible to estimate the accuracy and robustness of the algorithm in the presence of backgrounds with different characteristics. In particular, three scenarios with different types of periodic disturbances were simulated. The signal to be extracted, I(x), is for all three cases the same Lorentzian function written in the form of Equation (6) with $P_{1}=5\pi$ and $P_{2}=5$. All three scenarios were chosen to reflect real cases that are typical of TDLAS.

The first scenario is chosen to represent the experimental situation when the background has a periodic disturbance with an FSR comparable to the width of the line to be detected. This type of background is particularly problematic because it strongly affects the determination of the line shape. Furthermore, it cannot be removed by introducing a small jitter on the diode laser current and averaged out [29] or be filtered out with standard post-processing methods, without introducing a significant distortion of the line shape. This type of background is taken here as a simple a cosine function

(8) B(x)=0.07cos(0.1x+1).

**Figure 4:**
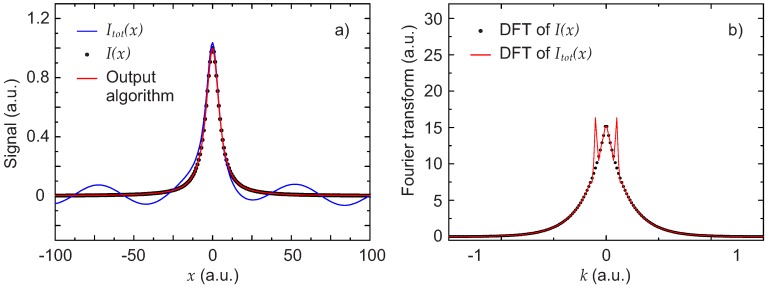
First scenario: disturbance with an FSR comparable to the line width. (**a**) simulated experimental total signal $I_{tot}\left(x\right)$ (blue line), Lorentzian line shape signal I(x) (black dots) and extracted signal obtained with the algorithm (red line); (**b**) DFT of the total signal $\left|F(I_{tot}\left(x\right))\left(k\right)\right|$ (red line) and DFT of the Lorentzian signal |F(I(x))(k)| (black points).

The total signal $I_{tot}\left(x\right)$ in this case, together with the expected signal I(x) are shown in Figure 4a. Also shown is the result obtained by applying the described algorithm. Despite the problematic background, the output of the algorithm is practically identical to I(x). The percent deviation of the two parameters $P_{1}$ and $P_{2}$ describing the Lorentzian obtained with the algorithm from the initial value used to generate I(x) is only of 0.27% for $P_{1}$ and 0.23% for $P_{2}$.

To better illustrate the contribution of the background, the DFT of the Lorentzian and the DFT of the total signal signal are also shown in Figure 4b. The two peaks visible in the figure represent the contribution of the background. With the pseudo-code algorithm described in Section 2.1, the cut-off was $i_{0}=13$.

The second scenario discussed here is that of a background with a weak disturbance characterized by an FSR much larger than the line width and as large as or larger than the measuring range. This type of disturbance arises because of reflections between two surfaces separated by a very short physical dimension, like the laser-chip output face and the glass window of the laser packaging. The chosen functional form to simulate this scenario is the following:

(9) B(x)=0.07cos(0.02x+1.6).

**Figure 5:**
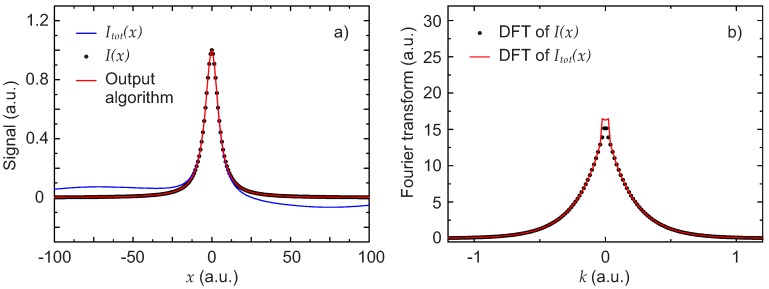
Second scenario: weak disturbance with an FSR as large as the measuring window. (**a**) simulated experimental total signal $I_{tot}\left(x\right)$ (blue line), Lorentzian line shape signal I(x) (black dots) and extracted signal obtained with the algorithm (red line); (**b**) DFT of the total signal $\left|F(I_{tot}\left(x\right))\left(k\right)\right|$ (red line) and DFT of the Lorentzian signal |F(I(x))(k)| (black points).

The result of the algorithm for this scenario is shown in Figure 5. Figure 5a plots the total signal $I_{tot}\left(x\right)$, the expected signal I(x) and the result obtained by applying the algorithm. Figure 5b shows the DFT of the Lorentzian and the DFT of the total signal. The two peaks, due to the contribution of the background, are now very close to zero, which makes the extraction particularly unproblematic. With the pseudo-code algorithm described in Section 2.1, the cut-off was $i_{0}=7$.

In this case, it is also clear from the figure that the algorithm extracts the signal exceedingly well. The percent deviation of the two parameters describing the Lorentzian obtained with the algorithm from the expected values is only of 0.28% for $P_{1}$ and 0.25% for $P_{2}$.

As a third scenario, a background resulting from the sum of a hundred cosine functions is considered. This less realistic case is chosen to demonstrate that, no matter how dramatic the interferences are, the algorithm can extract signal I(x) very well. In addition, this scenario illustrates the case when the functional form of the background is too complex to be included in a nonlinear fit of $I_{tot}\left(x\right)$. The background is thus written as

(10) $$B\left(x\right)=\sum_{i=1}^{100}A_{i}\cos\left(w_{i}x+\phi_{i}\right),$$

where $w_{i}$ is chosen randomly from a normal distribution with an average equal to zero and a standard deviation of 0.1, $\phi_{i}$ from a normal distribution with an average equal to zero and a standard deviation of 0.2, and $A_{i}$ from a normal distribution with an average equal to zero and a standard deviation of 0.03.

**Figure 6:**
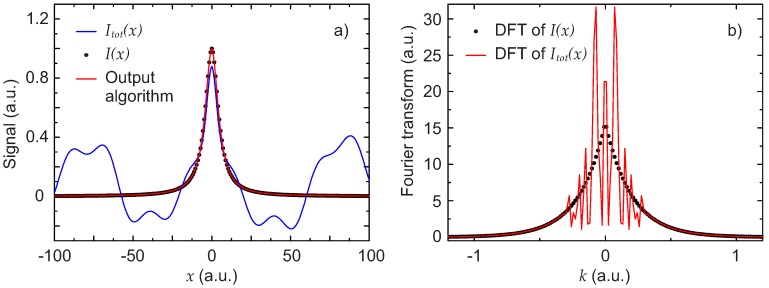
Third scenario: strong multiple disturbances. (**a**) simulated experimental total signal $I_{tot}\left(x\right)$ (blue line), Lorentzian line shape signal I(x) (black dots) and extracted signal obtained with the algorithm (red line); (**b**) DFT of the total signal $\left|F(I_{tot}\left(x\right))\left(k\right)\right|$ (red line) and DFT of the Lorentzian signal |F(I(x))(k)| (black points).

Figure 6a shows the total signal $I_{tot}\left(x\right)$, the expected signal I(x) and the result obtained by applying the described algorithm. Despite the very complicated background, the extraction of the signal I(x) by the algorithm works very well. In Figure 6b, the DFT of the Lorentzian and the DFT of the total signal are also shown. Due to the high number of cosine functions in the background, the DFT has a very structured shape. With the pseudo-code algorithm described in Section 2.1, the cut-off was $i_{0}=30$ (see also Figure 3).

The percent deviation of the two parameters describing the Lorentzian obtained with the algorithm for this scenario is only of 0.012% for $P_{1}$ and 0.14% for $P_{2}$. The deviation of both parameters is particularly low in this case. To better estimate the error and the standard deviation on the parameters, the method was applied to 500 functions created with the random sum of 100 cosines described above. Then, the error was evaluated and its distribution studied. As a result, $P_{1}$ has a mean value of the absolute value of the percentage error of 0.12% with a standard deviation of 0.19%, and $P_{2}$ a mean of 0.04% with a standard deviation of 0.06%.

Finally, the analysis of the algorithm with simulated data has allowed for determining the causes of the discrepancy between the parameters extracted with the algorithm and those of the starting function I(x). The main contribution to these arises from the approximation of the CFT by a DFT and is due to a rather large point spacing and limited x-range of I(x) used in the simulated data. Smaller contributions arise from an imperfect window compensation, and a very small frequency folding [30], which was neglected here. Since the purpose of this paper is to illustrate the algorithm and not to minimize the discrepancies, the simulated data were chosen to be as close as possible to typical experimental data. The application to the three scenarios demonstrate well how, even with a very complicated background like in the third one, the proposed algorithm can extract the underlying signal extremely well.

## 4. Experimental Results

To demonstrate the robustness of the method on real gas sensing measurements, absorption spectroscopy was performed on the three strong lines R9R9 (760.77 nm), R7Q8 (760.89 nm) and R7R7 (761.00 nm) of the O_2_ near infrared A-band in the presence of multiple interference fringes.

### 4.1. Experimental Setup

**Figure 7:**
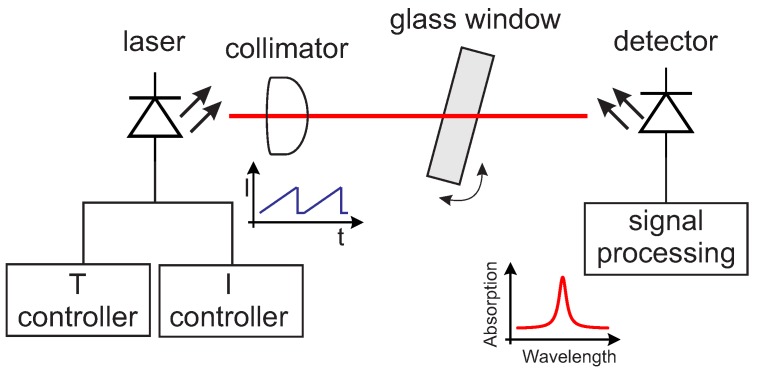
Schematic diagram of the setup used for the absorption spectroscopy experiments.

The setup for the absorption spectroscopy experiments was chosen to be extremely simple and is shown schematically in Figure 7.

The light source is a 0.25 mW single-mode vertical-cavity surface-emitting laser (VCSEL) (760 nm TO5 VCSEL, Philips Photonics, Ulm, Germany) emitting at 760 nm. The laser current and temperature were adjusted by a temperature controller (TEC 2000, Thorlabs, Newton, NJ, USA) and a VCSEL laser diode controller (LDC 200C, Thorlabs, Newton, NJ, USA). The light emitted by the laser is collimated by a lens. The light transmitted by the sample is collected using a large-area Si 10 × 10 mm_2_ photodiode (FS1010, Thorlabs, Newton, NJ, USA), amplified by an adjustable-gain photodiode amplifier (PDA200C, Thorlabs, Newton, NJ, USA). The current ramp for the wavelength-sweep and the data acquisition were performed by a DAQ card (USB-6361, National Instruments Switzerland GmbH, Ennetbaden, Switzerland) using a Labview^TM^ software. The laser current sweep was chosen so to be able to measure three oxygen absorption lines. The total distance between the laser and the detector was kept fixed at approximately 36 cm. Interference fringes of adjustable intensity were generated by inserting and tilting a glass window of known material in the optical path. By varying the thickness of the glass window, it is possible to achieve fringes with different FSR; by tilting the glass window, it is possible to adjust the amplitude of the fringes. In this work, two glass windows of BK7 of thicknesses d = 11 mm (window 1) and d = 4 mm (window 2) were used. These two windows were chosen to create interferences fringes with FSR comparable to (window 1) and greater than (window 2) the line width of the signal. These types of interferences are the most disturbing because they cannot be easily eliminated—for example, by a small jitter in the laser current or by standard post-processing filtering.

### 4.2. Oxygen Sensing

**Figure 8:**
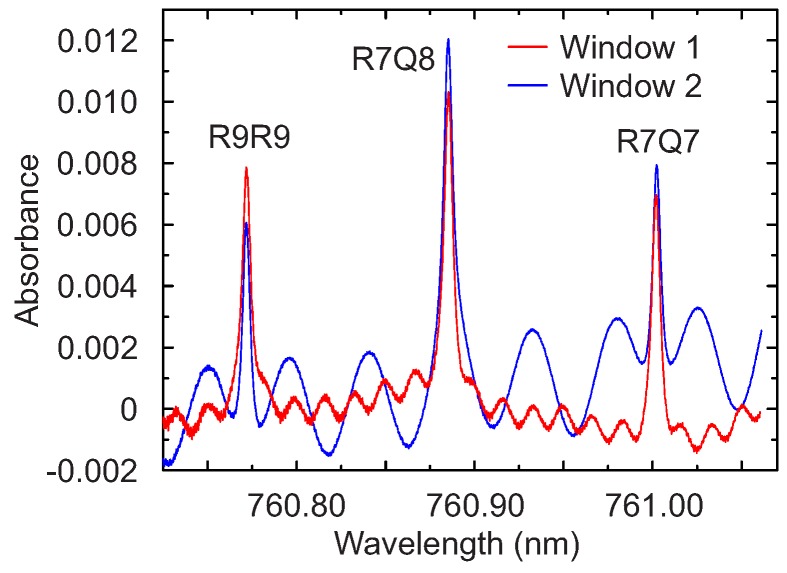
Typical absorbance for the three lines R9R9 (760.77 nm), R7Q8 (760.89 nm) and R7R7 (761.003 nm) of the O_2_ near infrared A-band. Superimposed to the absorption features are the interferences caused by the window 1 (d = 11 mm) and window 2 (d = 4 mm). The measurement is taken at atmospheric conditions.

The absorbance signal of the three oxygen lines R9R9, R7Q8 and R7R7 is shown in Figure 8. The interference fringes due to the glass window are clearly visible. Superimposed to these fringes, other minor ones are also visible, which are due to the glass window of the laser package and to the surfaces of the collimator. The zero-absorbance baseline arising from the nonlinear wavelength-dependent intensity of the laser was determined by a fourth-order polynomial fit over the entire sweep range. All the measurements reported in this work were performed in air, at room temperature and ambient pressure.

**Figure 9:**
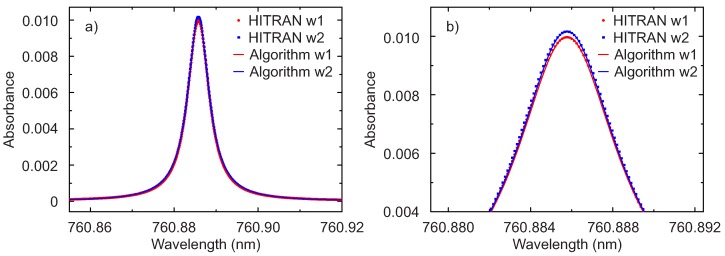
(**a**) comparison of the absorbance of the R7Q8 line extracted with the algorithm (solid lines; Algorithm w1, Algorithm w2) and the expected lines from HITRAN database (points; HITRAN w1, HITRAN w2) for the window 1 (w1, d = 11 mm) and window 2 (w2, d = 4 mm); (**b**) enlargement of the peak maximum for the same data.

Both measurements shown in Figure 8 were processed with the proposed algorithm assuming a Lorentzian line shape. The results for the line R7Q8 is shown in Figure 9. For comparison also the expected absorption lines based on the HITRAN 2012 database [31] and determined using the application SpectraPlot [32] are shown. It is evident from Figure 9, and particularly from the enlargement in (b), that the algorithm extracts the absorption line extremely well. Differently from many fitting methods, the algorithm does not require neither input parameters nor initial values. The slightly lower peak height for the line in the presence of window 1 is due to the fact that the distance between laser and detector was kept fixed and window 1 is thicker, thus reducing the optical path length for oxygen absorption of (11−4) mm =7 mm. This difference in the HITRAN simulated curves was extracted correctly by the algorithm. To estimate the accuracy of the algorithm, the area under the absorption line from the HITRAN database calculated numerically was compared with the value of $P_{1}$ extracted with the algorithm. For both measurements, the difference is 0.1%. There are still minimal deviations between the extracted and simulated lines, which could be reduced by considering a Voigt instead of a purely Lorentzian profile for the oxygen lines.

## 5. Conclusions

In this work, a novel semi-parametric algorithm is presented, which allows the extraction of a signal from an arbitrary background. In particular, the algorithm is applied to a background that is the sum of periodic interference fringes of different amplitudes and FSR. These types of disturbances, arising, for example, from multiple reflections between surfaces in the optical path, are of highest relevance for absorption spectroscopy because they are frequently the most common limit to gas sensor performance. The novel algorithm is firstly demonstrated on simulated data for three scenarios chosen to represent particularly relevant practical situations. In all three cases, the discrepancy between the results obtained with the algorithm and the expected values for the line parameters is very small—less than 0.3%. Then, the algorithm is applied to experimental data of oxygen absorption in the presence of multiple interferences. Despite the strong fringes, the extracted line shows a remarkable agreement with the expected curves from the HITRAN database, with deviation of the area of only 0.1%.

The great advantage of the algorithm is that it is semi-parametrical. This means that it requires no input parameters to extract the signal. This is clearly demonstrated by the third scenario with simulated data, where a background comprising a hundred cosine functions with different amplitudes, frequencies and phases could not be possibly modeled by standard nonlinear fits. In this work, the cut-off was determined dynamically by maximizing the coefficient of determination. Its recalculation at every measurement enables the accuracy achieved. This would be advantageous also in field applications, so that if the fringes should change in the time, the algorithm would automatically adjust the cut-off $i_{0}$.

These results show that the performance of a very simple sensing setup, with standard anti-reflection coatings and minimal precautions to minimize the interference fringes, can be strongly improved by simply post processing the data with the proposed algorithm.

In this paper, the algorithm was applied to a Lorentzian line shape in direct absorption spectroscopy for oxygen concentration determination. However, it can be applied to account for other line shapes, for example a Voigt profile, or to signals with a different functional form, as arising, for example, from wavelength-modulation spectroscopy. Particularly in the case of fringes with large amplitudes and FSR of the order of the FWHM of the line, it can extract the desired signal very well, making the signal insensitive to fringes and their changes with time. Preliminary results on simulated data indicate that the algorithm performs equally well even if the signal amplitude is ten times smaller than the fringes. Further studies are needed to test the performance of the algorithm in similar extreme cases.

In conclusion, the presented algorithm, being able to extract the signal feature from an arbitrary background, has the potential to allow interference-immune TDLAS, solving long-time stability problems arising from changes over time of the background, like thermal drift. Furthermore, this algorithm is not specific of TDLAS and can be applied to any kind of spectroscopic data, provided the functional shape of the signal to be detected is known.

## Abbreviations

The following abbreviations are used in this manuscript:

TDLAS	Tunable Diode Laser Absorption Spectroscopy
FSR	Free Spectral Range
CFT	Continuous Fourier Transform
DFT	Discrete Fourier Transform
RW	Rectangular Window
HWHM	Half Width at Half Maximum
VCSEL	Vertical-cavity surface-emitting laser

## Appendix A Approximation of the CFT by the Modified DFT

In this paper, the following definition for the continuous Fourier transform (CFT) is used

(A1) $$F\left(k\right)\equiv \int_{-\infty }^{\infty }f\left(x\right)e^{-ixk}dx.$$

Let us first make some assumptions that will simplify the calculations. Let us assume that the function f(x) is zero outside a certain range (−a/2,a/2) with some a∈R and a>0 and let us indicate with *m* the number of data points at our disposal. For convenience, *m* is taken to be even. Let us introduce the sampling rate β as

(A2) $$\beta =\frac{a}{m}.$$

The abscissas of the data $x_{j}$ and $k_{j}$ can be written as:

(A3) $$\begin{array}{cc} x_{j}= & \beta j, \end{array}$$

(A4) $$\begin{array}{cc} k_{j}= & \frac{2\pi }{a}j, \end{array}$$

with *j* going from −m/2 to m/2. We can approximate Equation (A1) with a Riemann sum using the fact that f(x) is zero outside the range (−a/2,a/2):

(A5) $$\begin{array}{cc} F(k_{n}) & =\int_{-\infty }^{\infty }f\left(x\right)e^{-ixk_{n}}dx\\ & =\int_{-a/2}^{a/2}f\left(x\right)e^{-ixk}dx\Rightarrow ,\\ F\left(k_{n}\right)=D_{n}\left(f\right) & \equiv \beta \sum_{j=-m/2}^{m/2}f\left(x_{j}\right)e^{-ix_{j}k_{n}}, \end{array}$$

where we have approximated the integral with a discrete sum. Clearly, the bigger *m* is, the better will be the approximation. Now, using Equations (A3) and (A4), we can rewrite (A5) as

(A6) $$D_{n}\left(f\right)=\beta \sum_{j=-m/2}^{m/2}f\left(x_{j}\right)e^{-2\pi ijn/m}.$$
